# Amyloid-Beta Related Angiitis of the Central Nervous System: Case Report and Topic Review

**DOI:** 10.3389/fneur.2014.00013

**Published:** 2014-02-04

**Authors:** Amre Nouh, Ewa Borys, Angelica K. Gierut, José Biller

**Affiliations:** ^1^Department of Neurology, Stritch School of Medicine, Loyola University Chicago, Maywood, IL, USA; ^2^Section of Neuropathology, Department of Pathology, Stritch School of Medicine, Loyola University Chicago, Maywood, IL, USA; ^3^Section of Allergy, Immunology and Rheumatology, Stritch School of Medicine, Loyola University Chicago, Maywood, IL, USA

**Keywords:** amyloid-beta related angiitis, primary angiitis of the central nervous system, cerebral amyloid angiopathy, leptomeningeal enhancement, cerebral amyloid angiopathy-related inflammation

## Abstract

Amyloid-beta related angiitis (ABRA) of the central nervous system (CNS) is a rare disorder with overlapping features of primary angiitis of the CNS and cerebral amyloid angiopathy. We evaluated a 74-year-old man with intermittent left sided weakness and MRI findings of leptomeningeal enhancement, vasogenic edema, and subcortical white matter disease proven to have ABRA. We discuss clinicopathological features and review the topic of ABRA.

## Case Report

**Figure 1:**
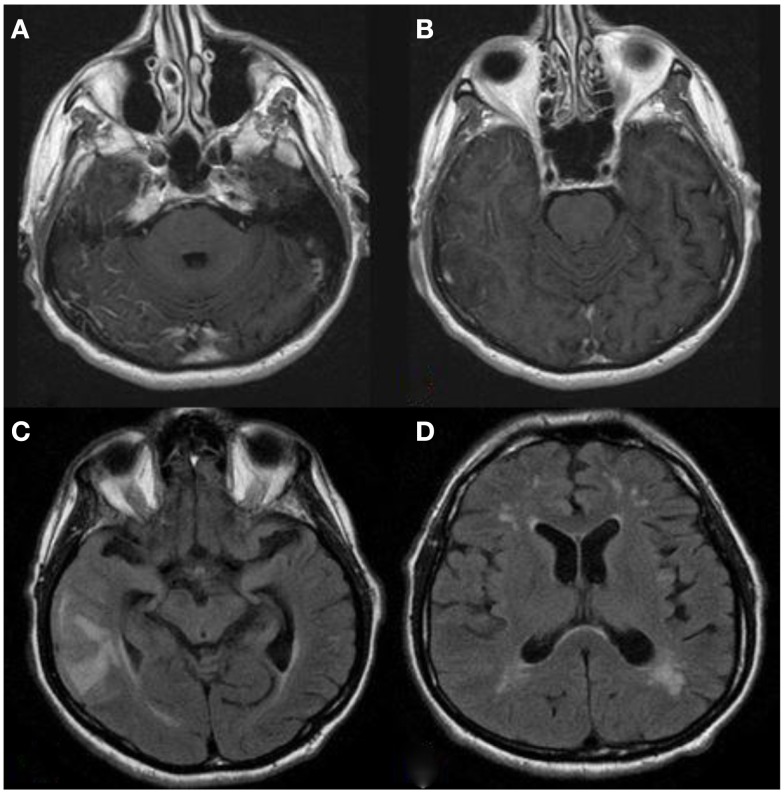
**(A,B)** T1 contrast-enhanced axial MRI shows evidence of right temporal leptomeningeal enhancement. **(C)** T2 FLAIR shows right temporal lobe vasogenic edema. **(D)** Scattered subcortical white matter disease.

**Figure 2:**
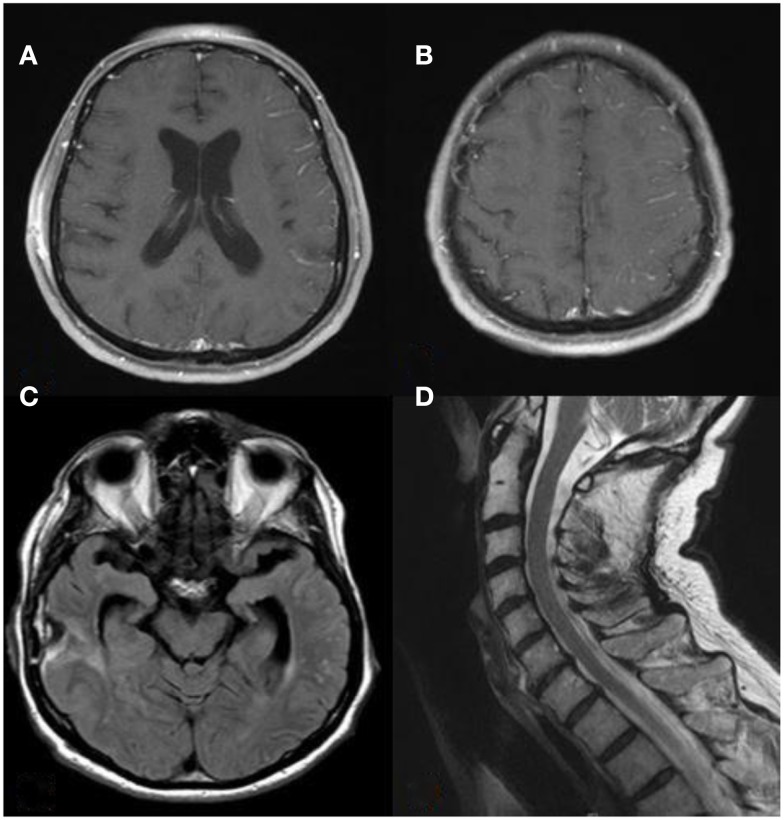
**(A,B)** T1 contrast-enhanced axial MRI shows new left frontal leptomeningeal enhancement. **(C)** T2 FLAIR shows new left temporal lobe changes. **(D)** T2 sagittal view of the cervical spine showed no evidence of intrinsic cord abnormalities.

A 74-year-old man with a past medical history of chronic lymphocytic leukemia (CLL) stage 0 (not receiving treatment), renal cell carcinoma (clear cell type, nuclear grade II-untreated), hypertension, and hyperlipidemia awoke one morning with numbness of the thumb and first two fingers of the left hand “marching” up to the left arm over the course of few seconds. This was immediately followed by drooling from the left corner of his mouth. The entire episode lasted <15 s and was followed by complete resolution. The following day, he had an episode of slurred speech and left facial weakness, which prompted evaluation at a local hospital where his symptoms again completely resolved. He was admitted for further investigation. Contrast-enhanced brain MRI showed leptomeningeal enhancement and increased signal intensity on T2 fluid attenuated inversion recovery (FLAIR) sequences on the right temporal lobe, as well as moderate subcortical and periventricular T2 signal abnormalities suggestive of white matter disease (Figures 1A–D). He was diagnosed as possibly having “herpes encephalitis” and intravenous Acyclovir was administered. Cerebrospinal fluid (CSF) analysis showed a white blood cell (WBC) count of 2 cells/μL, red blood cell count of 83 cells/μL, glucose of 61 mg/dL, and protein content of 282 mg/dL (normal range 15–45 mg/dL). Herpes simplex virus PCR was negative, and thus, Acyclovir was discontinued. Other laboratory analyses included an erythrocyte sedimentation rate (ESR) of 40 mm/h, a positive anti-nuclear antibody (titer unavailable), and negative anti-neutrophil cytoplasmic antibodies (ANCAs). During that hospital stay, he had several similar episodes of marching numbness involving his left hand and facial asymmetry without loss of consciousness or adventitious limb movements. Electroencephalogram (EEG) was normal. He received Levetiracetam 500 mg twice daily that was subsequently increased to 750 mg twice daily. He was discharged home without a definite diagnosis. However, the next day, he developed severe pain over the right temple and presented to another institution where a second MRI showed similar findings, and a right temporal leptomeningeal biopsy showed intramural as well as perivascular inflammatory infiltrates with granulomatous features in the leptomeninges and penetrating blood vessels associated with vasculopathic changes, as well as positive labeling for beta-amyloid on immunohistochemical studies consistent with amyloid-beta related angiitis (ABRA). He was then referred to our Medical Center for follow-up and management. Upon initial evaluation by us, neurologic examination was normal. However, 2 days later, he had mild right lower extremity weakness and slurred speech prompting hospital admission to our institution. A third brain MRI showed a new left frontal leptomeningeal area of enhancement and scattered FLAIR abnormalities on the left temporal lobe without evidence of acute infarction. MRI of the cervical spine showed no intrinsic cord changes (Figures 2A–D). EEG showed diffuse slow wave abnormalities. He received a 5-day course of 1000 mg intravenous methylprednisolone followed by a daily maintenance dose of 60 mg prednisone. Mycophenolate mofetil was chosen for immunosuppression in lieu of cyclophosphamide, given his CLL. The patient has remained neurologically stable since hospital discharge.

## Discussion

Amyloid-beta related angiitis is a predominantly granulomatous angio-*destructive* inflammatory mediated disease affecting leptomeningeal and cortical vessels characterized by meningeal lymphocytosis and abundant amyloid-beta deposition within the vessel walls (1, 2). Although sharing characteristics of both PACNS and cerebral amyloid angiopathy (CAA), ABRA has some differentiating clinical and pathological features. PACNS has an annual incidence of 2.4:1,000,000 and is diagnosed by the presence of acquired neurologic or psychiatric deficits and classic angiographic or histologic findings of CNS angiitis, unaccounted for by other systemic causes or disease mimics (3). CAA, characterized by beta-amyloid deposition (primarily the more soluble form of Aβ 1–40) within leptomeningeal and cortical vessels is found in 30–50% of asymptomatic elderly subjects and in approximately 80% of patients with Alzheimer’s disease. Moreover, CAA accounts for one third of all lobar hemorrhages in elderly subjects (4). A neuropathological variant of ABRA characterized by *non-destructive* perivascular inflammatory infiltration and amyloid deposition is known as CAA-related inflammation (CAA-RI) (5, 6). Patients with CAA-RI share most of the clinical features of ABRA, and most often present with seizures, and/or cognitive decline (5, 6). Both entities share similar clinical features that respond similarly to treatment (2).

The pathophysiology of ABRA is not fully understood. Whether amyloid-beta deposits initiate a vasculitic response or rather angiopathic-mediated inflammation leads to beta-amyloid deposition remains debatable. One theory suggests an immunologic response toward amyloid-beta, resulting in leptomeningeal and parenchymal inflammation with increased amyloid-beta deposition and clearance of amyloid-beta in the parenchyma (1). Decreasing levels of Aβ 1–40 and Aβ 1–42 CSF autoantibodies in two patients (one CAA-RI one ABRA) after steroid therapy further suggest an autoimmune response (2). The presence of partially activated CD4+ T-cells in the CSF (7), vasculitic changes in response to vascular amyloid in transgenic mice that develop CAA (8), and favorable response to steroids and immunosuppressive therapy supports an immune-mediated pathogenesis.

Amyloid-beta related angiitis usually presents in the seventh decade of life (mean age of 67 years) – without gender predilection (1). The main clinical features of ABRA, in order of frequency, include mental status and cognitive changes followed by headaches, seizures, and focal neurologic deficits (1, 2, 9, 10). In addition, patients may rarely present with uveomeningitis (11), posterior reversible encephalopathy-like manifestations, (10) or progression to coma (12).

An abnormal ESR has been reported in 12–38% of patients with ABRA (1, 2). In the series by Scolding et al., none of the 21 patients tested had positive ANCAs. In contrast, CSF abnormalities are extremely common and present in 85% of patients. Protein content elevation has been found in 60–90% of patients, with a median level of 100 mg/dL. Primarily, lymphocytic pleocytosis with a WBC count >5 cells has been observed in 50% of cases (1, 2). CSF oligoclonal bands have been absent in all patients tested by Scolding et al. (1). In one report, CSF tau and Aβ 42 were found to be normal in one patient with positive oligoclonal bands (13). CSF flow cytometric analyses have shown predominantly CD4+ T-cells (7). The most common APOE genotype found when compared to non-inflammatory CAA patients was APOE4, present in 71–76% of tested patients (5, 6).

The most common neuroimaging findings of ABRA are gadolinium-enhancing leptomeninges (2). Other findings include non-specific white matter changes, vasogenic edema, mass lesions, infarctions, and/or intracerebral hemorrhages (1, 2, 9, 10). Leptomeningeal or cortical hemosiderin deposits are observed in some cases (5, 7, 14) but unlike CAA, cerebral hemorrhages are less frequent (1, 2). Angiographic abnormalities are also infrequent (2, 5). Most common EEG abnormalities are diffuse slow wave changes (1).

Treatment of ABRA and CAA-RI include corticosteroids and immunosuppression, primarily with cyclophosphamide (1, 2, 5, 6). In one series, 5/6 patients with CAA-RI clinically improved with treatment, returning to their premorbid status. Four out of these five patients demonstrated MRI improvement, and only two deaths were reported between 2 and 3 years from initial presentation (5). In another series of 12 patients with CAA-RI, 7 improved within 1–2 weeks of treatment, 3 patients had relapses while not receiving treatment, and 2 patients showed no clinical response (6). In the series by Salvari et al., 10/28 patients with ABRA were treated with steroids alone, and 13/28 patients were treated with a combination of steroids and immunosuppression for 5–8 months (nine cyclophosphamide, four other agents). Those treated with steroids + immunosuppression fared better than patients with CAA but not differently than patients with PACNS. In the series by Scolding et al., approximately one in four patients required a surgical intervention such as CSF diversion, mass lesion resection, or craniotomy. Mortality in ABRA has been reported to be between 7 and 44% (1, 2).

Amyloid-beta related angiitis and CAA-RI are unusual neuropathological diagnoses. Mental status or cognitive changes, gadolinium-enhancing meninges, and elevated CSF protein content are frequent findings. In contrast to PACNS, patients with ABRA have better response to immunosuppressive therapy. Differentiating prognostic and management features between ABRA and CAA-RI require further refinement.

## Conflict of Interest Statement

The authors declare that the research was conducted in the absence of any commercial or financial relationships that could be construed as a potential conflict of interest.

## Figures and Tables

**Figure A1:**
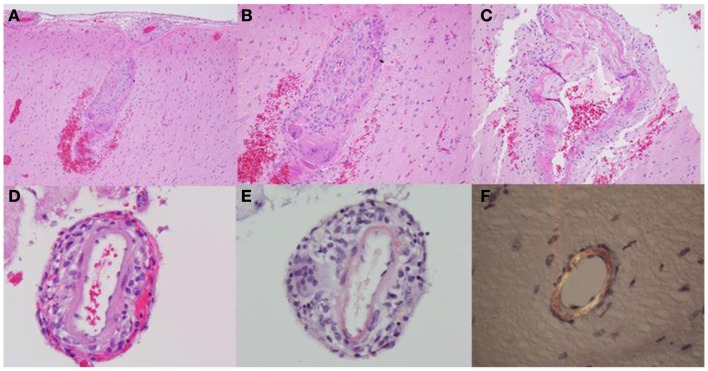
**(A,B)** Leptomeningeal and cortical blood vessels with granulomatous inflammation and surrounding hemorrhage, 40× and 100×. **(C)** Cortical vessel with inflammation and fibrinoid necrosis, 200×. **(D)** Vessel with granulomatous inflammation and amyloid deposition, 400×. **(E)** Congo red stain positive for amyloid, 400×. **(F)** Congo stain showing characteristic apple-green birefringence under polarized light, 400×.

## Appendix

### Pathology

Brain biopsy showed cortical and subcortical white matter with perivascular chronic intramural inflammatory infiltrates with fibrinoid deposition along with granulomatous features involving meningeal and superficial penetrating arteries. In many instances, the inflammatory changes were associated with deposition of amorphous eosinophilic material in the vessel walls consistent with amyloid. Congo red and β-amyloid immunohistochemical stain were positive.
